# Chemical injuries of the oesophagus: aetiopathological issues in Nigeria

**DOI:** 10.1186/1749-8090-4-56

**Published:** 2009-10-16

**Authors:** Martins O Thomas, Ezekiel O Ogunleye, Oladapo Somefun

**Affiliations:** 1 Lagos University Teaching Hospital/College of Medicine of University of Lagos, Nigeria

## Abstract

**Background:**

Chemical injuries of the oesophagus occur worldwide. There is paucity of information on aetiopathological profile of chemical injuries of the oesophagus in Nigeria.

**Aim:**

The aim of the study was to determine the aetiopathological pattern of chemical injuries of the oesophagus in Nigeria.

**Materials and methods:**

This is a multi-centre hospital based study in Lagos metropolis spanning a period of 10 years.

The patients' bio data, substances ingested, sources of corrosives, reasons for ingesting corrosives and patients' mental state were recorded.

**Results:**

In all, there were 78 patients (61 Males, 17 Females). The offending agents were acids in 55.1% of cases and it was accidental ingestion in 62 patients. The highest incidence of 57.6% was found in the middle 1/3 of the oesophagus.

**Conclusion:**

Accidental ingestion of acids is the commonest cause of oesophageal injuries in Nigeria. The incidence of severe strictures necessitating oesophageal substitution could be reduced if early management of corrosive oesophagitis improves in Nigeria.

## Introduction

Chemical injuries of the oesophagus are caused by ingestion of corrosives like acids, alkali and some neutral substances. Other causes of oesophagitis include autoimmune diseases, infection, radiation and gastro-oesophageal reflux disease (GERD) [1-13].

Ingested corrosives produce oro-pharyngeal and gastro-oesophageal injuries ranging from minor burns to severe necrosis, depending on the agent, the quantity ingested, concentration and duration of exposure. This may lead to corrosive strictures of the oesophagus [2].

Various aspects of corrosive strictures of the oesophagus have been studied worldwide. A report from Ibadan, in South-Western Nigeria, by Ajao, OG and Solanke, TF [3] concluded that the commonest cause of benign oesophageal stricture is ingestion of corrosives.

Acids tend to burn the oral cavity, pharynx or larynx at the upper end and the pylorus is often damaged with copious ingestion of acids. Attempt at spiting out the acid may lead to tell-tale signs on the skin of the chest.

Alkalis tend to affect the body of the oesophagus, especially at areas of natural constrictions. Powdery substances like calcium carbide do not glide easily in the oesophagus, so they tend to cause local damage which often leads to perforation.

The pathology of oesophageal injuries has been studied extensively using animal and human models. The pathology is broadly divided into acute and chronic phases for description.

### Acute Phase

It is important to note that even though acute inflammatory reaction is found in the acute phase irrespective of the causative agent [6]. In animal models, most authors agree to the presence of intraepithelial segmented leucocytes, sub epithelial leucocytes, basal cell hyperplasia and ulcers depending on the depth of mural involvement. In severe cases, wall perforation may lead to mediastinitis.

This histological pattern forms the basis for adoption of Dameron and Wangensteen classification of jejunal injuries [6] to score oesophageal injuries as below.

A score of 1 is for necrosis that is limited to mucosa. Sub-mucosal involvement attracts a score of 2; involvements of muscularis propria, adventitia or outright perforations are scored 3, 4 and 5 respectively.

The scoring system for peptic injury of the oesophagus is fairly different. Grading of gastro oesophageal reflux diseases (GERD) is as follow:

Grade I: this is a non specific oesophagitis. In addition to neutrophilic infiltration, biopsy shows hyperplasia of basal layers.

Grade II: There is breakdown of mucosa and frank ulceration.

Grade III: Grade II plus attempts at healing (laying down of granulation tissue).

Grade IV: This is reflux-induced oesophageal stricture.

Grade IV may also be associated with or preceded by cephalad migration of squamo-columnar junction for more than 3 cm, a situation of Barrett's oesophagus is diagnosed. This is a pre-malignant lesion.

### Late Phase

This is the phase of established stricture. It develops when the acute phase is not well managed [6].

There is progressive cicatrisation of the offended segment of the oesophagus leading to stricture formation. In a living person, contrast oesophagogram and oesophagoscopy will show the number, length and calibre of the stricture(s).

In a 14-year review by Osinowo O and Alonge T [4] in Ibadan, South Western Nigeria, corrosive strictures constituted 17.4% of the indications for oesophageal replacement among the 47 patients studied.

For surgical purposes, it is good to classify strictures as dilatable or non dilatable. Dilatable strictures are single short segment strictures (<2.5 cm) with residual lumina that can take bougie dilators.

Non dilatable strictures are the multiple or long segment strictures.

Complete loss of lumen falls in this category no matter the length.

As their names suggest, dilatable strictures can be opened up by forceful passage of dilators or bougies through them. The undilatable strictures are only amenable to surgery [7].

Short segment non dilatable strictures can be cured by resection and re-anastomosis. Long segment strictures with residual lumina can be treated by patch oesophagoplasty while others are treatable by oesophageal substitution. From the foregoing, it is clear that knowledge of the aetiology and pathology of chemical burns of the oesophagus will ultimately determine the applicable treatment modality. All the methods throw up different treatment challenges in this part of the world.

This study was conducted to highlight the aetiopathological pattern of chemical injuries of the oesophagus in Nigeria.

## Patients and methods

This is a prospective study of patients who reported with features suggestive of chemical injuries of the oesophagus from June 1996 - May 2006. It is a multi-centre, hospital based study in Lagos Metropolis in Nigeria. The study was conducted in 4 centres. A protocol was designed and the needed data were carefully entered. The data collected included patients' ages, gender, the corrosives ingested if any, the source(s) and reasons for ingesting the corrosives and patients' mental state. Hypotheses were formulated as follows:

Ho: Location of stricture is independent of acid or alkali ingestion. H1: Location of stricture is not independent of acid or alkali ingestion.

During the first contact, the time lag from injury to presentation was documented. Patients were asked questions relating to the management of the acute phases of their injuries. Specifically, the mode of treatment was documented vis-à-vis the attempt at inducing emesis before presenting at the hospitals, use of antidotes like oil, water or other specific antidotes (like acid for alkali and alkali for acids).

Endoscopic findings at the acute phase were noted. Questions were asked about specific use of naso-gastric tubes, antacids or H_2 _blockers, and steroids as part of the initial management at the referring hospital.

The patients had barium swallow to determine the segment affected the length, calibre and number of strictures. We relied on history of peptic ulcer disease and/or gastro-oesophageal reflux preceding dysphagia for the diagnosis or peptic strictures.

Biopsy samples got at endoscopy or surgical specimens of late cases were sent for histology. The results of which were documented.

For descriptive purposes, multiple strictures affecting one segment were taken as one entity. In the same vein, long strictures affecting two oesophageal segments were taken as two. A measure of dispersion of the distribution was carried out.

We sought correlation between age and number of strictures using Pearson Correlation Coefficient. We used chi-square to test the significance of acids and alkali's preference for upper 1/3, middle 1/3 and lower 1/3 strictures using two by three contingency tables.

We sought correlation between age and incidence of strictures using the Pearson Product Moment Correlation Coefficient and their coefficient of determination was also found.

## Results

A total of seventy eight patients were studied within the 10-year period. This comprised of 61 males and 17 females giving a M:F ratio of 4:1. Patients in the 20 - 39 year age range were 42, constituting 53.8% of the series while 31 (39.7%) were 10 years and below. (Table 1).

**Table 1:** Age and Sex Distribution of the patients

**Age (Yrs)**	**M**	**F**	**Total**
< 10	10	8	18(23.1%)
10-19	7	6	13(16.7%)
20-29	23	2	25(32.1%)
30-39	16	1	17(21.8%)
40-49	2	-	2(2.6%)
> 50	3	-	3(3.8%)

Total	61(78.2%)	17(21.8%)	78(100.0%)

The mean age of the patients was 22.56+ 2.89 years at α 0.05 (δ = 13.03 years). The median age was 23.3 years. The distribution enjoyed a Pearson's Skewness of -0.15 and it was leptokurtic.

Regression analysis of age (x) against the number of cases (y) brought up a linearity of Y = 22.942 - 0.3314x.

The offending substances were acids (battery water) in 43 patients (55.1%) and alkali in 28 patients (35.9%) (Table 2). Attempted suicide was the reason for ingestion in 8 patients while it was accidental in 62 others. Parents of seven of the children stored caustic soda for making soap while others got corrosives from different sources. One patient was forced by armed robbers to drink an unknown substance. One patient, who was depressed, mixed cement with battery acid in a suicide attempt (Table 2). None of the other patients had overt psychiatric disturbances [12].

**Table 2:** Showing aetiology and number of victims

**Aetiology**	**No of victims**
Acids	43(55.1%)
Alkali	28 (35.9%)
Drugs	1 (1.3%)
Peptic Strictures	5 (6.4%)
Unknown Substance	1 (1.3%)

Total	78(100.0%)

A total of six patients presented in early phase for early endoscopy. The findings in them were oral burns in four and mid oesophageal grade II burns in the remaining two. A total of forty seven patients attempted emesis in the acute phase, 12 had specific treatments including the use of naso-gastric tubes, antacids or H_2 _blockers and/or steroids in the acute phase. In 27 cases, non specific use of palm oil was applied at home in acute phase supposedly to neutralize the causative agent. None of the patients was given water in the acute phase as part of pre-hospital treatment.

Peptic strictures accounted for 5(6.4%) cases. There were three pharyngo-oesophageal strictures, three in upper third, 57 in middle third and 29 lower third strictures. The stomach was involved in 7 patients (Table 3). In all, there were 99 strictures in 78 patients. Statistical analysis showed no correlation between age and number of strictures.

**Table 3:** Aetiology and Location of Strictures

**Aetiology**		**No and location of strictures**				
	**Pharyngo-oesophageal**	**Upper 1/3**	**Middle 1/3**	**Lower 1/3**	**Stomach**	**Total**

Acids	3	2	23	17	7	52(52.5%)
Alkali	-	1	33	6	-	40 (40.4%)
Drugs	-	-	1	-	-	1(1.0%)
Peptic	-	-	-	5	-	5(5.1%)

Total	3(3.0%)l	3(3.0%)	57(57.6%)	29(29.5%)	7(7.1%)	99(100.0%)

All the strictures involving the pharyngo-oesophagus and stomach were caused by acids. In the same vein GERD caused some lower 1/3 strictures. Using chi-square for assessment of the location of strictures caused by acids and alkalis (*χ^2 ^calculated *> *χ^2 ^table at α 0.01*) we rejected Ho.

It is important to note that the only drug-induced stricture was found in a young adult who used a herbal preparation to treat his tooth ache. His father had to stop this treatment when he noticed the onset of dysphagia in the child.

All the adult patients were in social class IV and below. None of them worked with acids so there was no occupational predisposition.

When correlation was calculated between the age and number of cases, the Pearson Product Moment Correlation coefficient r was -0.69. R2 (coefficient of determination) evaluation showed that only 47.61% of the cases could be explained with this correlation.

## Discussion

Oesophagitis, corrosive or non corrosive occur worldwide except for the relative preponderances of causative agents in different localities. The important agents earlier documented in this locality by Odelola and her colleagues [10], while reviewing 24 paediatric cases were caustic soda (75% of cases) and acids. Adegboye and his colleagues in Ibadan, South Western Nigeria had 22 patients who developed strictures following ingestion of corrosives in five years.

More conclusively, majority of our patients (55.1%) ingested acids (battery water) while 35.9% ingested alkali-mainly caustic soda.

The reversal of alkali preponderance in reported paediatric series [10] could be explained by the fact that children will only ingest what is readily available to them. In Nigeria, caustic soda is used and kept mostly by parents who make soap. Therefore, caustic soda is more available to the children of such parents.

On the other hand, battery water is handled mainly by car battery technicians who use sulphuric acid as electrolytes. These electrolytes are usually kept in their workshops where they are not readily available for children to swallow.

Another important factor is that acids taste sour; therefore, children who swallow acids may not be able to take enough volume to cause major strictures.

It is significant to note that in our series, there was no occupational predisposition to strictures.

It is also important to note that only six patients presented early enough for early endoscopy. The corollary here is that most of our patients either presented late in the acute phase or they came with established strictures.

The large number of patients who induced emesis before presenting brings to fore, the level of ignorances as to the danger posed by such practice in worsening the pathology of corrosive burns of the oesophagus. The outward passage of the corrosive may have worsened the ensuing strictures in them.

Treatment of acute chemical burns of the oesophagus is still less than optimal in Nigeria. In our series, only 12 patients had specific treatment in the acute phase. Many patients still believe the old practice of drinking palm oil to neutralize whatever corrosive agent that is swallowed. It is obvious that most people are not aware of the beneficial effect of drinking water especially in cases of accidental ingestion of corrosives. Water is more readily available at home than specific antidotes. Its use may improve pre-hospital treatment of oesophageal burns in the acute phase.

The case of a patient that was forced by armed robbers to drink an unknown substance introduced a new dimension to criminal activities in Nigeria. From our local experience, though not published, robbers usually kill their victims either for lack of cooperation of the victims or when there is risk of disclosure of identity of the robbers by the victims. In our practice, this is the first contact with a patient with this type of experience where the victim was maimed by forceful ingestion of corrosive.

We saw an unusual drug induced stricture in a young adult who used a herbal preparation to cure his toothache. The depressed patient who mixed cement with corrosive is another strange case.

It is our conclusion that the male patients still maintained their dominance over females in the incidence of corrosive strictures in Nigeria and corrosive ingestion is still mostly accidental among Nigerian adults. The occurrence of severe strictures, necessitating major operations like oesophageal substitution could be reduced if management of corrosive oesophagitis is well carried out at primary and secondary levels of care. The pre-hospital care of such patients may also improve the outlook. It is important to reiterate the call for enforcement of existing laws controlling the use of chemicals in Nigeria. This may reduce the availability of corrosives either for accidental or deliberate consumption.

## Competing interests

The authors declare that they have no competing interests.

## Authors' contributions

MO performed all the procedures as lead surgeon, he designed the study, wrote the manuscript and performed the statistical analysis, EO assisted MO during most of the procedures, and AO assisted MO for the operation of difficult strictures.
